# Notice of Mr. James Gardette's "Observations on the Transplantation of Teeth," &c.

**Published:** 1850-07

**Authors:** E. B. Gardette


### ARTICLE II.

*Notice of* MR. JAMES GARDETTE'S "*Observations on the Transplantation of Teeth,*" *&c.* BY E. B. GARDETTE, M. D.

The re-publication in the "American Journal and Library of Dental Science," for January, 1850, of the above paper, written in 1827 by my father, and then published in the "Medical Recorder," of Philadelphia, has naturally claimed my attention with something more than the ordinary interest of a general reader. The "Observations," of course, are not new to me, unless in that sense of novelty in which a forgotten object re-appears before the mind in a new form and under changed circumstances; and with whatever feelings of politeness the contributor and editors may have offered or received the paper for the Journal, it seems to me of doubtful utility or compliment to professional readers of the present time. Such, I feel sure, would have been the opinion of the writer himself, if now living, and I think he would not willingly have consented to the re-publication, without at least subjecting the "Observations" to appropriate revisal.

And yet, even with this feeling, or impression, however strongly entertained, I could not well intrude the offer of such editing, previous to the insertion in the Journal, although kindly notified by the Baltimore Editor of the intention to re-publish the paper. The gentleman who contributed it is an entire stranger to me, but I owe him the courtesy of my thanks for the evident kindness with which he desired to revive and preserve the "Observations," whilst I am induced now to notice them with perchance some degree of filial and familiar freedom, for which the profession will scarcely demand of me an apology.

In reporting the unsuccessful cases of transplanted teeth by Mr. Lemayeur, and the comparatively successful ones by the celebrated John Hunter, that chanced to come under Mr. Gardette's notice, there seems a want of proper care on his part, in making the obvious distinctions that bear upon the question he had under consideration, or in arriving at correct conclusions from their peculiarities. These oversights arose, I do not doubt, from the great reluctance, the aim at brevity, and "the diffidence *he* always felt in writing English for publication;" and if I am able, in any degree, to supply some of the deficiencies just referred to, I shall not merely have performed a grateful duty towards the memory of my father and preceptor, but possibly carry out more fully his own views, as he would have had them understood.

I proceed to notice the omissions, and what seem to me the objectionable portions of the "Observations."

We are told that "*Mr. W. H. of Philadelphia had three upper incisors transplanted in London in* 1784–5," and that "*five years afterwards,*" Mr. Gardette was first consulted on account of their looseness: and that by relieving these same teeth from the mechanical action of the opposite incisors, and by the use of astringent washes, "*they again became firm in the course of two weeks.*" It would be important and interesting to know how long they *continued firm,* after the relief thus afforded by Mr. Gardette.

Another patient, we are informed, also had an upper front incisor transplanted by Mr. Hunter in 1780, who still had the tooth in 1783, and that this tooth was removed in consequence of a discharge of pus, "*of which the lady did not notice the progress for some years.*"

These two cases (and the *only* two performed under Mr. Hunter's superintendence,) were attended with a degree of success which contrasts sadly with the total failure of "*one hundred and seventy cases of transplanted teeth by Mr. Lemayeur,*" and "*more than fifty of which*" were extracted by Mr. Gardette within a brief period afterwards.

The widely different character of the *results* seem calculated to raise a doubt whether Mr. Lemayeur deserved "the reputation of an eminent dentist" ascribed to him; for he appears to have been eminently unsuccessful in the experiment of transplanting teeth, scattering dead bones upon his path through the land *at five guineas* a piece. At all events, I am naturally led to make some distinctions between the skill and judgment exercised in the performance of the operations by the two gentlemen, (Mr. Hunter and Mr. Lemayeur,) and also as to the constitutional differences in the individuals upon whom they operated. These striking contrasts would also evidently suggest more extended and pathological conclusions than the mere summary fact that "*the transplanted tooth will not become firm and useful.*" It is to be regretted that Mr. Gardette, while engaged with the subject, did not give to the profession a more detailed account of his views respecting causes as well as consequences, his own *rationale,* derived from knowledge and observation.

An eloquent medical lecturer of Baltimore, in one of his many happy spontaneous efforts of wit and learning, proved satisfactorily to his hearers that there is often too much importance attached to the knowledge supposed to be derived from experience. Unfortunately, I do not possess the means of quoting the language of the able medical man referred to, but from the recollection of his views I borrow the thought, that in surgery as well as in medicine, we are too apt to believe we know a fact from experience, when, in truth, we have only been misled into conclusions more or less correct, by a few experiments. Now the experience of Mr. Gardette, in regard to the transplantation of teeth, would have been rendered especially valuable by the correct reflections and deductions he was abundantly able to make upon what he had seen; for his knowledge, I may safely assert, was much greater than his experience, upon this particular subject. From the latter we only learn that over fifty teeth out of one hundred and seventy, transplanted by Mr. Lemayeur, were failures, and extracted by him within a very short period after the operations of transplanting; but what became of the remaining one hundred and twenty teeth transplanted by the same gentleman, his experience tells us nothing about.

The two cases from London that came under the observation of Mr. Gardette, seem to have had almost an encouraging degree of success, as respects the number of years they remained in the mouth; but with what amount of comfort, purity, health or usefulness, the observations do not inform us.

The opinion that the operation is impracticable, or, in the language of the "Observations," that "*there are a thousand chances to one against the success of transplanting teeth from one mouth into another*," however just or a good guide with reference to the inducements to attempt it, cannot be regarded as satisfactorily explained or sustained by the cases reported in Mr. Gardette's paper. Nor is the theory a sound one upon which the whole objections to the operation are founded, viz: "that the root of the tooth which is to replace the defective one should be of the same *length, size and shape* of the root of the one which is to be replaced, and that the dentist is obliged to judge of this without seeing either," for this permits the conclusion that if, by a very possible chance, the roots of two teeth should so precisely resemble each other "*length, size and shape,*" that then the operation of transplanting teeth from one mouth into another, would be entirely successful. Or, in other words, that the mechanical fit or adjustment of the fang of the new tooth, to the form of the socket from which a tooth had just been removed, is the sole principle upon which the success or failure of the transplantation of teeth depends. Such surgical operations are not to be estimated or determined upon by a calculation of chances, and this was by no means, I am quite confident, the precise meaning Mr. Gardette intended to convey, and certainly not all he might have written as to the principles involved. He was well aware, of course, that the unavoidable destruction of the periostiums of the fang and alveolus, and the necessary rupture of the dental membranes at the apex of the fang, resulting from the extraction of a tooth, are most important considerations in estimating the propriety or success of transplanting teeth from one mouth to another. Upon the health of these depend the life, the living principle of the tooth, surrounding parts, and, as we all know, it is their disturbance and the consequent diseased action that renders the operation impracticable.

A dead tooth introduced into the living jaw, must continue to act upon the adjacent parts as an irritating foreign body, and the degree of mischief it occasions will be governed by the greater or less amount of natural excitability of temperament, or the accidental condition of the circulation at the time. It is well ascertained that there are constitutional peculiarities or idiosyncrasies, differing so materially and strangely from one another in different individuals, as to render what would prove to be a fatal freedom with the frame in one case, harmless and even comfortable in another. The natural or physiological laws upon which the health and usefulness of the human teeth depend, may not be violated to the extent of transplanting a dead tooth in the living jaw, without the penalties of inflammation, suppuration, morbid or active disease in some form: the light or serious character of these will, in each case, be determined by the favorable circumstances, or otherwise, in regard to constitution, and the extent of local disturbance inflicted.

It is something less formidable, to be sure, to plant another tooth in the head, and expect it to grow, than it would be to place a "new head upon old shoulders," after removing the defective incumbrance that might call for exchange; but mostly it would be wiser for the patient who is about to submit to a change of teeth by transplantation, to make a change of heads in his dentist. I do not mean by the same operation, for even the nice surgery of Saladin's sabre, that took off the head of the Grand Master of the Templars so quickly that the trunk remained standing, could not transplant another in its place.

As to the operation of restoring a tooth to its original socket, after partial or entire extraction, it can scarcely be termed transplantation of a tooth; and although such a tooth also loses its vitality, the exactness with which it fills its own alveolar cavity, and the slow progress of ensuing symptoms, are the chief senses in which it differs in condition from a tooth transplanted into another mouth. The sundering and absorption of the nerve and periostiums; the loss of all connection with the general circulation; all the objectionable features, though less aggravated, of a transplanted tooth, are likely to supervene, if a tooth is replaced after having been extracted. Hence, inducements can rarely exist for making such an experiment as the restoration of a tooth, once entirely removed from its alveolus; for the extension of inflammation and diseased action upon neighboring teeth and gums, involves premature loss or injury. Such doubtful operations, to say the least, seem a heavy penalty for the abortive effort to improve looks for a brief space, at the risk of serious derangement of health, and the loss of that comfort to be derived from even a diminished number of sound and unmolested teeth.

Doctor Eleazar Parmly has said that "a tooth is worth bearing just so much pain," and I think that a restored or a transplanted one is not worth the endurance of any pain at all: and by this rule, it were not difficult, perhaps, to classify the value of teeth in the mouths of different individuals. To the young, for instance, a troublesome, unseen molar, is not worth a moment's inconvenience: while a front, or side tooth, that "shows," is worth years of martyrdom. And thus it has been that transplanted front teeth have been endured in the mouth with all the evils attendant upon their presence, especially at a period of time when artificial teeth were not very successfully supplied. The experiment of transplanting teeth from one mouth to another, at the risk of health and even life, seems somewhat characteristic of the age and country in which it originated, where, under the *ancien règime*, a lady would stand in starch and buckram, bedaubed with paint, powder and pomatum for a whole day, in order to figure at a ball in full costume at night.

Mr. Gardette's observations bear strong testimony in favor of the disputed theory that the most revolting diseases may be introduced from one system to another by the transplantation of teeth; and the most eminent medical men have frequently been deceived by remote symptoms that eventually have been traced to dead or diseased teeth, long after having had recourse in vain to general remedies for that most indefinite of maladies—neuralgia. The stomach, and other guiltless organs, are often thus punished for the sins of some vile hidden fang, or diseased tooth, whose slow, deep-seated action, nevertheless, produces the distant but acute paroxysm. The diseases, especially, of the antrum maxillare, have not been sufficiently observed by the general medical practitioner, in their connection with diseased teeth, and I use this occasion to express my great pleasure at the expectation of a highly valuable work on this subject, with numerous illustrations, by Dr. Edward Maynard, of Washington City. This gentleman deserves great credit for his extensive cabinet, collected in Europe, and through which he will be able to develope many original and interesting facts to the profession.

Before closing this desultory notice of the "Observations on the Transplantation of Teeth," I earnestly disclaim all affinity or sympathy with that "march of mind" in modern times, which goes to prove conclusively that sons know much more than their sires; but if, in the performance of my voluntary duty, I have been able to give any value to this paper, or through it to add in the least to the force of that from the pen of my father, I trust it may be regarded as only an attempt to restore to his memory a small part of the advantages I have derived from his teachings and character.

---

